# Chromium-Doped Biomass-Based Hydrochar-Catalyzed Synthesis of 5-Hydroxymethylfurfural from Glucose

**DOI:** 10.3390/polym17101413

**Published:** 2025-05-20

**Authors:** Huimin Gao, Wei Mao, Pize Xiao, Chutong Ling, Zhiming Wu, Jinghong Zhou

**Affiliations:** Guangxi Key Laboratory of Clean Pulp & Papermaking and Pollution Control, School of Light Industrial and Food Engineering, Guangxi University, Nanning 530004, China; gaohuimmm@st.gxu.edu.cn (H.G.); maowei@st.gxu.edu.cn (W.M.); 2216301047@st.gxu.edu.cn (P.X.); lingchuttt@st.gxu.edu.cn (C.L.); 2216391048@st.gxu.edu.cn (Z.W.)

**Keywords:** biomass, hydrothermal carbonization, hydrochar, chromium doping, 5-Hydroxymethylfurfural

## Abstract

5-Hydroxymethylfurfural (HMF) is a versatile carbohydrate-derived platform chemical that has been used for the synthesis of a number of commercially valuable compounds. In this study, several chromium (Cr)-doped, biomass-derived hydrochar catalysts were synthesized via the one-pot method using starch, eucalyptus wood, and bagasse as carbon sources. Then, the performance of these synthesized materials for the catalytic conversion of glucose into HMF was evaluated by, primarily, the yield of HMF. The synergistic interactions between the Cr salt and the different biomass components were investigated, along with their effects on the catalytic efficiency. The differences in the catalytic activity of the synthesized materials were analyzed through structural characterization, as well as assessments of the acid density and strength. Among the catalysts, Cr_5_BHC_180_ derived from bagasse presented the highest activity, achieving an HMF yield of 64.5% in an aqueous solvent system of dimethyl sulfoxide (DMSO) and saturated sodium chloride (NaCl) at 170 °C after 5 h. After four cycles, the HMF yield of Cr_5_BHC_180_ decreased to 38.7%. Characterization techniques such as N_2_ adsorption–desorption and Py-FTIR suggested that such a decline in the HMF yield is due to pore blockage and acid site coverage by humic by-products, as demonstrated by the fact that regeneration by calcination at 300 °C restored the HMF yield to 50.5%.

## 1. Introduction

5-Hydroxymethylfurfural (HMF) is a key intermediate for the production of many valuable compounds including 2,5-dimethylfuran and furan-2,5-dicarboxylic acid via hydrogenation and oxidation reactions, owing to its reactive functional groups of a furan ring, hydroxyl, and formyl [1,2]. The synthesis of HMF can be accomplished by dehydrating biomass-derived carbohydrates, including fructose, glucose, starch, and cellulose [3,4]. Among these carbohydrates, glucose—the most abundant natural hexose—can be obtained from cellulose and starch through acidic or enzymatic hydrolysis. [5] The production of HMF from glucose is generally composed of two key processes: (1) isomerizing glucose into fructose, which requires a structural shift from pyranose to furanose that can generally be achieved by catalysis using Lewis acids or bases as catalysts [6], and (2) dehydrating fructose into HMF, which can be accelerated with Brønsted acid sites [7]. Of the two steps, isomerization is generally considered the rate-limiting step [8].

Metal-ion-loaded catalysts are especially effective for the above-mentioned transformation of glucose to fructose. For its properties of high thermal stability, porosity, large surface area, and environmental compatibility, biochars can serve as an excellent support for such catalysts [9]. Hydrochars prepared from biomass via hydrothermal carbonization possess abundant oxygen-containing groups and acidic sites [10,11]. Many studies have shown that embedding metal salts in situ during hydrothermal treatment enables the uniform distribution of metal nanoparticles on the surface of hydrochars, enhancing the performance of the catalyst supported by hydrochar [12]. This improvement is attributed to the introduction of Lewis acid sites by doped metal ions, which promote fructose dehydration through Brønsted acid catalysis. For instance, Liu et al. synthesized a nano-aluminum-doped hydrochar from corn stover, achieving a fructose yield of 42.6% and glucose selectivity of 83.6% at 180 °C within 5 min [13]. Similarly, Kang et al. developed Mg-Al-K-C-modified hydrochars from cellulose, creating diverse catalytic sites—including Lewis acids and Brønsted bases—for efficient glucose isomerization [14]. Additionally, Syed M. prepared aluminum oxide-silica/carbon composites from rice husk, which acted as bifunctional Lewis–Brønsted acid catalysts, delivering an HMF yield of 52% in N-methylpyrrolidone at 170 °C [15]. Beyond these metals, Sn, Cr, Fe, and others have also been widely applied in glucose to HMF conversion systems [16]. Notably, chromium (Cr) functions effectively as a Lewis acid on its own and can synergize with other catalysts, such as solid acids, to further enhance selectivity and yield. For example, Rakngam et al. loaded various metal phosphates (MPO, M = Cr, Zr, Nb, Sr, Sn) onto Al-SBA-15 for glucose conversion, with CrPO_4_/Al-SBA-15 exhibiting the highest catalytic activity, attributed to its superior total acidity and optimal Lewis-to-Brønsted acid site ratio [17]. Similarly, Perez et al. developed three solid SSA-based catalysts—Ti-SSA, Zr-SSA, and Cr-SSA—using SSA as an organic ligand, with Cr-SSA proving the most effective for glucose isomerization, ultimately achieving an HMF yield of 60% [18].

In this study, starch, eucalyptus, and bagasse were selected as representative biomass feedstocks for the preparation of three different types of hydrochars, from which and from chromium(III) chloride (CrCl_3_), three Cr-doped hydrochar catalysts were prepared via a one-pot hydrothermal synthesis for the catalysis of glucose into HMF. Then, the impacts of CrCl_3_ doping on the hydrochar catalysts were comprehensively investigated in terms of their product yield, surface morphology, functional groups, degree of carbonization, acid strength and density, and the existence of Lewis acid and Brønsted acid sites. Moreover, the performance of the as-prepared hydrochar catalysts was tested in a dimethyl sulfoxide (DMSO)/sodium chloride (NaCl) aqueous solvent system. Additionally, an evaluation was performed regarding the influences of the reaction conditions of temperature and time during catalysis with the Cr-doped catalysts on the yield of HMF, along with the recyclability of the catalysts.

## 2. Materials and Methods

### 2.1. Materials and Chemicals

Bagasse was sourced from Guangxi Guitang (Group) Co., Ltd., Guigang, China. and eucalyptus was supplied by a local tree farm in Guangxi, China. Both the materials were milled and then sieved through a sieve (60 mesh) before use. Soluble starch (99%), CrCl_3_·6H_2_O (99%), D-(+)-glucose, 5-HMF (99%), NaCl (99.5%), DMSO (99.8%), methanol (chromatography reagent), and acetic acid (anhydrous) were obtained from Shanghai Aladdin Biochemical Technology Co., Ltd., Shanghai, China. Sulfuric acid (98%) was obtained from Tianjin Fuyu Fine Chemical Co., Ltd., Tianjin, China. Unless otherwise specified, the water used was deionized water prepared in-house. All other materials and chemicals were analytically pure and directly used without additional treatment.

### 2.2. Preparation of Biomass-Derived Hydrochar Catalysts

For synthesis of the catalysts, 4 g of each carbon source material (starch, eucalyptus, and bagasse), 5 g of CrCl_3_·6H_2_O, and 40 mL of water were thoroughly mixed and then hydrothermally treated at 180 °C for 4 h in a 100 mL reactor. Afterward, the product was cooled to room temperature, followed by thorough washing with water, filtration, and, finally, drying at 80 °C for 8 h to obtain the respective Cr-doped catalyst. The control samples were prepared in the same way in the absence of the use of CrCl_3_·6H_2_O.

The as-synthesized Cr-doped hydrochar catalysts were designated as Cr_5_SHC_180_ (starch-based), Cr_5_EHC_180_ (eucalyptus-based), and Cr_5_BHC_180_ (bagasse-based), respectively, and the respective undoped hydrochars, i.e., the control samples, were labeled as Cr_0_SHC_180_, Cr_0_EHC_180_, and Cr_0_BHC_180_.

### 2.3. Characterization

The characterization of the as-prepared catalysts was performed, in terms of the elemental composition and chemical state of the surface, by X-ray photoelectron spectroscopy (XPS, Thermo Scientific K-Alpha, Waltham, MA, USA); the crystal structure, using X-ray diffraction (XRD, MiniFlex600, Rigaku, Tokyo, Japan); the specific surface area and pore size distribution, by N_2_ adsorption-desorption isotherms (Micromeritics ASAP 2460, Norcross, GA, USA); the structural transformation, by laser Raman spectroscopy (InVIA Reflex, Renishaw, Gloucestershire, UK); the surface morphology, by scanning electron microscopy (SEM, ZEISS Sigma 300, Oberkochen, Germany); and the nature and density of acid sites, by pyridine-adsorbed Fourier-transform infrared spectroscopy (Py-FTIR, Nicolet iS50, Thermo Fisher Scientific, Waltham, MA, USA), with pyridine adsorption performed at 180 °C after evacuation at 200 °C for 1 h.

### 2.4. Catalytic Conversion of Glucose to HMF

Glucose underwent catalytic reactions using each the catalysts in a Teflon-lined stainless steel autoclave (Thermo Fisher Scientific, Waltham, MA, USA) placed in a high-temperature oven. For each catalytic reaction, 0.5 g of glucose and an appropriate amount of the catalyst were mixed with 10 mL of the solvent (a mixture of 9 mL of DMSO and 1 mL of saturated NaCl solution). The autoclave was sealed and then heated to 140, 150, 160, 170, and 180 °C for a specified duration (1–5 h). Afterward, the autoclave was placed into an ice bath to rapidly cool to 20–35 °C. Finally, the product was subjected to centrifugation at 9000 rpm for 5 min, and then the supernatant was collected and analyzed by high-performance liquid chromatography (HPLC).

### 2.5. Analysis of Catalytic Products

The catalytic products were analyzed on a Waters E2695 HPLC (Waters, Singapore) for the concentrations of glucose, fructose, and HMF. For glucose and fructose, the analysis was conducted using a refractive index detector and a Bio-Rad Aminex^®^ HPX-87H column (7.8 mm 300 mm; Bio-Rad Laboratories, Hercules, CA, USA), 5 mmol/L sulfuric acid as the mobile phase, and the following chromatographic conditions: a flow rate of 0.6 mL/min, and a column temperature of 50 °C. For HMF, the analysis was carried out using a UV detector and a ZORBAX Eclipse XDC-C18 column (4.6 × 250 mm, 5 µm, Element Lab Solutions, Strathaven, UK), a mixture of 1% acetic acid (anhydrous) and methanol (chromatography grade) (90:10 (*v*/*v*)) as the mobile phase, and the following chromatographic conditions: the detection wavelength of 285 nm, the column temperature of 30 °C, the detector temperature of 40 °C, and the flow rate of 1.0 mL/min. Additionally, the injection volume for both the above two analyses was the same as 20 µL of each the collected supernatant filtered using a 0.22 µm membrane filter. Based on the results of HPLC analyses, the amounts in moles of unreacted glucose, obtained fructose, obtained HMF, and original glucose can be calculated, and then glucose conversion (%), fructose yield (%), HMF yield (%), and HMF selectivity (%) can be obtained according to the following formulas:

(1) $${\mathrm{Glucose}}_{\mathrm{conversion}}=\frac{\mathrm{amount}\;(\mathrm{moles})\;\mathrm{of}\;\mathrm{reacted}\;\mathrm{glucose}}{\mathrm{amount}\;(\mathrm{moles})\;\mathrm{of}\;\mathrm{original}\;\;\mathrm{glucose}}\times 100\%$$

(2) $${\mathrm{Fructose}}_{\mathrm{yield}}=\frac{\mathrm{amount}\;(\mathrm{moles})\;\mathrm{of}\;\mathrm{obtained}\;\mathrm{fructose}}{\mathrm{amount}\;(\mathrm{moles})\;\mathrm{of}\;\mathrm{original}\;\mathrm{glucose}}\times 100\%$$

(3) $${\mathrm{HMF}}_{\mathrm{yield}}=\frac{\mathrm{amount}\;(\mathrm{moles})\;\mathrm{of}\;\mathrm{obtained}\;\mathrm{HMF}}{\mathrm{amount}\;(\mathrm{moles})\;\mathrm{of}\;\mathrm{original}\;\mathrm{glucose}}\times 100\%$$

(4) $${\mathrm{HMF}}_{\mathrm{selectivity}}=\frac{{\mathrm{HMF}}_{\mathrm{yield}}}{{\mathrm{Glucose}}_{\mathrm{conversion}}}\times 100\%$$

## 3. Results and Discussion

### 3.1. Characterization of Hydrochar Catalysts

#### 3.1.1. XPS Analysis

**Figure 1:**
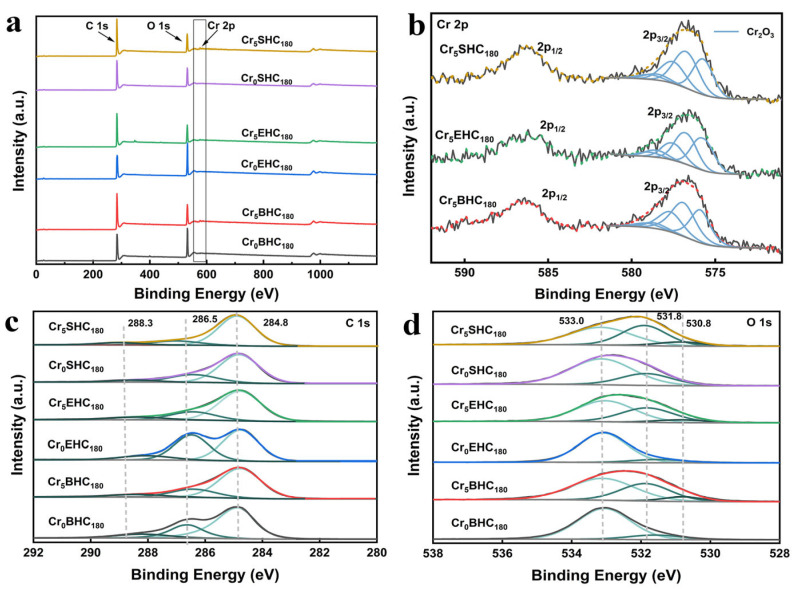
XPS spectra of different biomass-based hydrochar catalysts: (**a**) full spectra, (**b**) Cr 2p spectra, (**c**) C 1s spectra, (**d**) O 1s spectra.

- Figure 1 presents the XPS spectra of all the hydrochar catalysts derived from starch, eucalyptus, and bagasse. As shown in Figure 1a, all Cr-doped catalysts of Cr_5_SHC_180_ (starch-based), Cr_5_EHC_180_ (eucalyptus-based), and Cr_5_BHC_180_ (bagasse-based) contain Cr, C, and O elements. The Cr 2p spectra (Figure 1b) display two peaks at 586.9 and 577.4 eV, which can be assigned to Cr2p_1/2_ and Cr2p_3/2_ of Cr(III) [19], respectively. Deconvolution of the Cr 2p_3/2_ peaks reveals five sub-peaks at 576.5, 577.5, 578.3, 579.3, and 579.7 eV, which are indicative of the presence of Cr_2_O_3_ [20], confirming successful loading of chromium oxide on all of the hydrochar catalysts.
- As shown in Figure 1c, the C 1s spectra show the peaks that can be attributed to C-C/CHx/C=C (284.6 ± 0.2 eV), C-OH/C-O-C (285.7 ± 0.2 eV), and C=O (287.3 ± 0.2 eV) [21]. In the O 1s spectra provided in Figure 1d, there are the peaks that can be assigned to C-OH/C-O-C (533.0 ± 0.2 eV), C=O (531.8 ± 0.2 eV), and Cr-O (530.8 ± 0.2 eV) [22]. In comparison to the respective undoped counterparts, all of the Cr-doped catalysts showed a significantly reduced content of C–OH groups on their surfaces, particularly Cr_5_SHC_180_ and Cr_5_BHC_180_. Such reduction is attributed to the enhanced acidity due to the introduction of CrCl_3_ during hydrothermal synthesis. Bagasse, with a higher hemicellulose content (23–27%) and lower lignin content (19–32%) than eucalyptus (hemicellulose: 18–23%; lignin: 29–33%) [23], was more reactive. When CrCl_3_ is present, the metal ions disrupt the fibrous structure of biomass by osmosis, catalyzing the dehydration and decarboxylation of α-glycosidic bonds in starch and hemicellulose [22]. As illustrated in Table 1, the content of C–OH on the surface was markedly decreased from 72.65 to 50.41 for Cr_5_SHC_180_, from 86.92 to 57.14 for Cr_5_BHC_180_, and from 89.03 to 57.21 for Cr_5_EHC_180_. Additionally, the C–C/CHx/C=C peak intensities are increased significantly, indicating that chromium salts can enhance the aromatization process during hydrothermal carbonization [24].

#### 3.1.2. XRD and Raman Spectral Analyses

**Figure 2:**
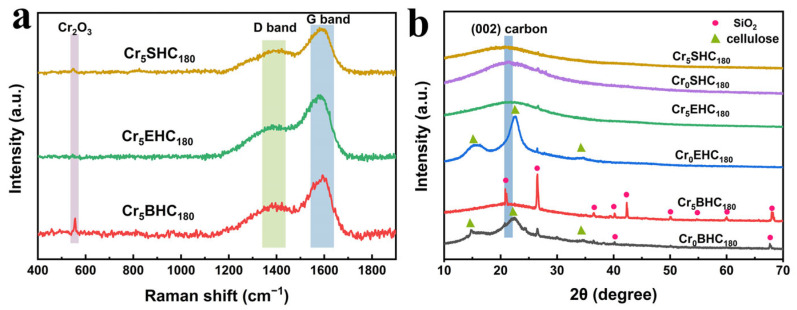
(**a**) Raman spectra and (**b**) XRD spectra of different biomass-based hydrochar catalysts.

The carbonization characteristics of Cr-doped hydrochar catalysts were analyzed by Raman spectroscopy and XRD. As shown in Figure 2a, the Raman spectra display a D band (1325–1350 cm^−1^), representing structural defects in carbon atoms, and a G band (1580–1610 cm^−1^), which can be assigned to the in-plane stretching vibrations of sp^2^-hybridized carbon atoms [25]. A signal at 556 cm^−1^ can be attributed to Cr_2_O_3_, indicating the successful incorporation of Cr^3+^ into the hydrochar matrixes [26,27], which is in agreement with the results of the Cr 2p XPS spectra (Figure 1b). The intensity ratio of the D and G bands (I_D_/I_G_) for Cr_5_SHC_180_, Cr_5_EHC_180_, and Cr_5_BHC_180_ was 1.33, 1.86, and 1.27, respectively. Since the larger the I_D_/I_G_ ratio is, the more structural defects and the lower the degree of graphitization [28], Cr_5_BHC_180_ exhibited a higher degree of carbonization than both Cr_5_SHC_180_ and Cr_5_EHC_180_. Notably, as shown in Figure 2a, the Cr_2_O_3_ signal at 556 cm^−1^ is absent for Cr_5_EHC_180_, likely due to the fact that its higher lignin content may have masked the Cr_2_O_3_ peak within the broad carbon background [22]. As shown in Figure 2b, the XRD patterns of Cr_0_EHC_180_ and Cr_0_SHC_180_ display characteristic cellulose peaks at 2θ = 14.8, 22.7, and 34.5° [29], indicating incomplete carbonization. In contrast, all Cr-doped catalysts exhibited a broad peak centered at 2θ = 23.7°, characteristic of amorphous carbon [30,31], suggesting enhanced carbonization compared to the undoped samples. This implies that metal salt doping accelerates the aromatization process during hydrothermal carbonization. No distinct peaks for crystalline chromium oxides were detected, likely due to the uniform dispersion of amorphous Cr_2_O_3_ within the carbon matrix, which prevented the formation of detectable crystalline domains [32]. Additionally, characteristic SiO_2_ was observed in the bagasse-based hydrochar, attributed to silicate absorbed by sugarcane during growth [33].

#### 3.1.3. SEM Morphological Analysis

**Figure 3:**
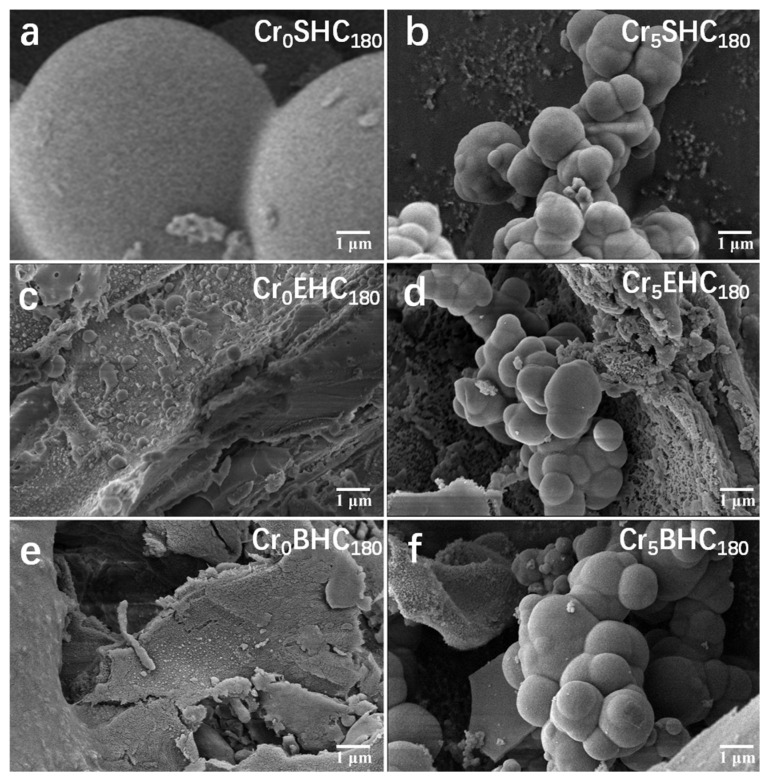
SEM images of different biomass-based hydrochar catalysts. (**a**) Cr_0_SHC_180_; (**b**) Cr_5_SHC_180_; (**c**) Cr_0_EHC_180_; (**d**) Cr_5_EHC_180_; (**e**) Cr_0_BHC_180_; (**f**) Cr_5_BHC_180_.

The examination of the surface morphology of the biomass-based hydrochar catalysts was conducted by SEM, and the results are provided in Figure 3. As shown in Figure 3a,b, the starch-derived hydrochar without CrCl_3_ (Cr_0_SHC_180_) exhibits spherical particles with an average diameter of approximately 7.15 µm, while upon the addition of CrCl_3_, the particle size is significantly decreased to 1.07 µm. Such reduction is due to the coordination of Cr^3+^ ions with hydroxyl and other oxygen-containing groups in starch, which forms an intermolecular cross-linked network that inhibits starch chain expansion and restricts the growth of carbon spheres [34]. The hydrochars derived from eucalyptus (Cr_0_EHC_180_) and bagasse (Cr_0_BHC_180_) without the addition of CrCl_3_ maintained relatively intact fibrous structures, while the ones (Cr_5_EHC_180_ and Cr_5_BHC_180_) with the addition of CrCl_3_ displayed a typical hydrothermal carbon morphology, i.e., carbon skeletons embedded with microspheres. Notably, Cr_5_BHC_180_ showed larger microspheres than Cr_5_EHC_180_. This difference is likely due to the higher hemicellulose content in bagasse, which, under the catalytic effect of Cr^3+^, undergoes accelerated isomerization and dehydration, promoting the nucleation and growth of hydrochar microspheres [22,35]. In contrast, lignin contributes minimally to microsphere formation, and a higher lignin content may even hinder microsphere development [22]. Additionally, the N_2_ adsorption–desorption analysis (Figure S1) and Table 2 indicate that the presence of CrCl_3_ during hydrochar synthesis provided the catalyst a higher specific surface area. According to Table 2, with the addition of CrCl_3_, the yield of hydrochar from starch is significantly increased from 4.61% to 18.06%, while the yields from both eucalyptus and bagasse are decreased from 60.72% to 36.36% and 29.12% to 23.96%, respectively.

#### 3.1.4. Thermal Stability Analysis

**Figure 4:**
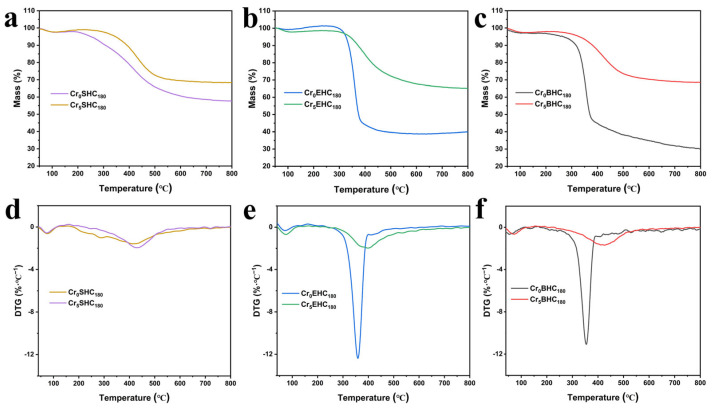
TG curves of (**a**) starch-based, (**b**) eucalyptus-based, and (**c**) bagasse-based hydrochars; DTA curves of (**d**) starch-based, (**e**) eucalyptus-based, and (**f**) bagasse-based hydrochars.

- The hydrochar catalysts were analyzed for their thermal stability by thermogravimetric (TG) and differential thermal analysis (DTA), and the results are presented in Figure 4, from which it is clear that both the bagasse- and eucalyptus-derived hydrochars follow similar thermal degradation patterns consisting of three main stages: dehydration, devolatilization, and combustion [36]. The thermal stability of the hydrochars is significantly enhanced with the incorporation of Cr. For the eucalyptus-based hydrochar, the temperature corresponding to the maximum degradation rate is increased from 359 °C to 400 °C, while mass loss is decreased from 60% to 36%. Similarly, in the bagasse-based hydrochar, the peak degradation temperature is shifted from 355 °C to 426 °C, and the mass loss drops from 70% to 32%. In the case of starch-based hydrochar, the maximum degradation temperature is slightly increased from 422 °C to 436 °C and the mass loss is reduced from 32% to 18% with Cr doping. All of these results demonstrate that the incorporation of Cr increases the resistance of the catalyst to thermal degradation, thereby offering improved thermal stability.

### 3.2. Catalytic Performance of Hydrochar Catalysts

#### 3.2.1. Effects of Acid Density and Strength on Catalytic Performance

**Figure 5:**
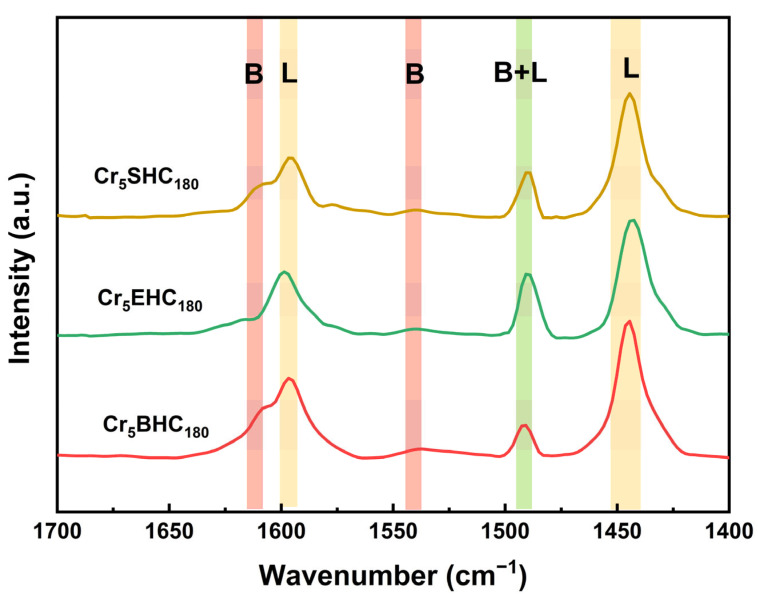
Py-FTIR spectra of different biomass-based hydrochar catalysts.

- The impact of the incorporation of Cr on the acid density and strength of the catalysts is summarized in Table 3. For the undoped catalysts of Cr_0_SHC_180_, Cr_0_EHC_180_, and Cr_0_BHC_180_, the initial potentials are 221, 159, and 225 mV, respectively, with corresponding acid densities of 0.14, 0.10, and 0.16 mmol/g. Upon Cr doping, the respective values were increased significantly to 247, 164, and 265 mV, and to 0.20, 0.18, and 0.24 mmol/g. Among the catalysts, Cr_5_BHC_180_ exhibited the highest acid strength and density, correlating with its superior catalytic performance, with both increased HMF yield from 36.7% (undoped) to 52.2% (doped) and improved HMF selectivity from 40.2% (undoped) to 52.2% (doped). In contrast, Cr_5_EHC_180_ showed the lowest HMF yield, attributed to its comparatively lower acid strength and density [37]. This enhancement of acidic functionality directly influences the catalytic activity, as demonstrated by the trends of increasing glucose conversion, fructose yield, and HMF yield and selectivity with acid strength and density [38,39]. The types of acid sites and the amounts of acid in the prepared catalysts were determined using Py-FTIR (Figure 5). Specifically, Cr_5_BHC_180_ had the highest concentration of Brønsted acid (4.11 μmol g^−1^) and Lewis acid (31.86 μmol g^−1^), with a concentration ratio of Brønsted acid to Lewis acid of 0.13. In comparison, the Brønsted acid concentrations for Cr_5_SHC_180_, and Cr_5_EHC_180_ were 2.46 and 2.84 μmol g^−1^, and the Lewis acid concentrations were 28.65 and 29.62 μmol g^−1^, with a respective concentration ratio of Brønsted acid to Lewis acid of 0.09 and 0.10. All of these results confirm that Cr_5_BHC_180_ possesses the highest acidity and, thus, the highest catalytic efficiency for converting glucose into HMF.

The strength of the initial potential [40]: −100~0 mV for weak acids, 0~100 mV for strong acids, 100 mV or above for super strong acids; reaction conditions: 0.5 g of glucose; 0.1 g of the catalyst; 10 mL of DMSO/NaCl_aq_ (9:1 (*v*/*v*)); 180 °C; and 4 h.

#### 3.2.2. Influence of Reaction Conditions on Catalytic Performance

**Figure 6:**
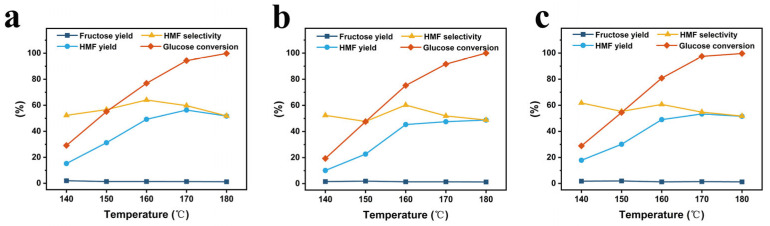
Effect of reaction temperature on HMF yield and selectivity for (**a**) Cr_5_SHC_180_, (**b**) Cr_5_EHC_180_, and (**c**) Cr_5_BHC_180_. Reaction conditions: 0.5 g of glucose, 0.1 g of catalyst, and 10 mL of DMSO/NaCl_aq_ (9:1, *v*/*v*); temperatures of 140 °C, 150 °C, 160 °C, 170 °C, and 180 °C; reaction time of 4 h.

**Figure 7:**
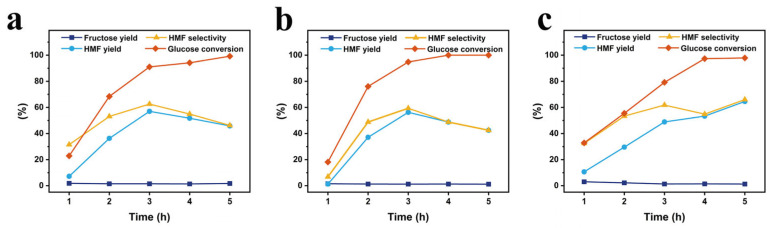
Effect of reaction time on HMF yield and selectivity for (**a**) Cr_5_SHC_180_, (**b**) Cr_5_EHC_180_, and (**c**) Cr_5_BHC_180_. Reaction conditions: 0.5 g of glucose, 0.1 g of catalyst, and 10 mL of DMSO/NaCl_aq_ (9:1, *v*/*v*); temperatures: (**a**) 170 °C, (**b**) 180 °C, and (**c**) 170 °C; reaction times of 1, 2, 3, 4, and 5 h.

- The influence of the conditions of reaction temperature and reaction time on the catalytic performance of the catalysts was evaluated with respect to the HMF yield, HMF selectivity, fructose yield, and glucose conversion. For the reaction temperature, the analysis was performed at varying temperatures of 140 °C, 150 °C, 160 °C, 170 °C, and 180 °C with all other reaction conditions the same, and the results are provided in Figure 6. From the figure, it is evident that at 140 °C, 150 °C, 160 °C, 170 °C, and 180 °C, the HMF yields are 15.2%, 31.2%, 49.2%, 56.3%, and 51.7%, respectively, for the starch-based Cr_5_SHC_180_; they are 10.1%, 22.6%, 45.3%, 47.5%, and 48.8% for the eucalyptus-based Cr_5_EHC_180_; and 17.8%, 30.1%, 49.0%, 53.3%, and 51.5%, respectively, for the bagasse-based Cr_5_BHC_180_. With the increase in the reaction temperature, the glucose conversion rate also increased from 20.0% to 99.6%, and the fructose yield remained stable at 1.5%. In all cases, the HMF yield presented a trend of increasing at first and then decreasing with temperature due to side reactions like HMF rehydration and condensation after reaching certain high temperatures, and the selectivity of HMF also decreased [41]. As a result, the optimal reaction temperatures for maximum HMF yield were identified as 170 °C for Cr_5_SHC_180_ and Cr_5_EHC_180_, and 180 °C for Cr_5_BHC_180_.

- For the reaction time, the experiments were performed at the respective optimal temperature for each the catalysts and the reaction times of 1, 2, 3, 4, and 5 h, with all other reaction conditions the same, and the results are depicted in Figure 7. As indicated in the figure, the HMF yields at 1, 2, 3, 4, and 5 h are 7.2%, 36.3%, 57.0%, 51.7%, and 45.8%, respectively, for the starch-based Cr_5_SHC_180_; 1.24%, 37.14%, 56.2%, 48.8%, and 42.5%, respectively, for eucalyptus-based Cr_5_EHC_180_; and 10.7%, 29.6%, 48.9%, 53.3%, and 64.5%, respectively, for the bagasse-based Cr_5_BHC_180_. The optimal reaction times and performance metrics for each catalyst were as follows: (1) Cr_5_SHC_180_: at 170 °C for 3 h, the HMF yield was 57.0%, HMF selectivity was 62.6%, glucose conversion was 91.0%, and fructose yield was 1.5%; (2) Cr_5_EHC_180_: at 180 °C for 3 h, the HMF yield was 56.2%, HMF selectivity was 59.3%, glucose conversion was 94.8%, and fructose yield was 1.2%; (3) Cr_5_BHC_180_: at 170 °C for 5 h, the HMF yield reached 64.5%, with an HMF selectivity of 66.0%, glucose conversion of 97.8%, and fructose yield of 1.3% (the HPLC chromatograms of the final HMF detection of the catalysts are shown in Figures S2 and S3).

#### 3.2.3. Catalyst Recyclability

**Figure 8:**
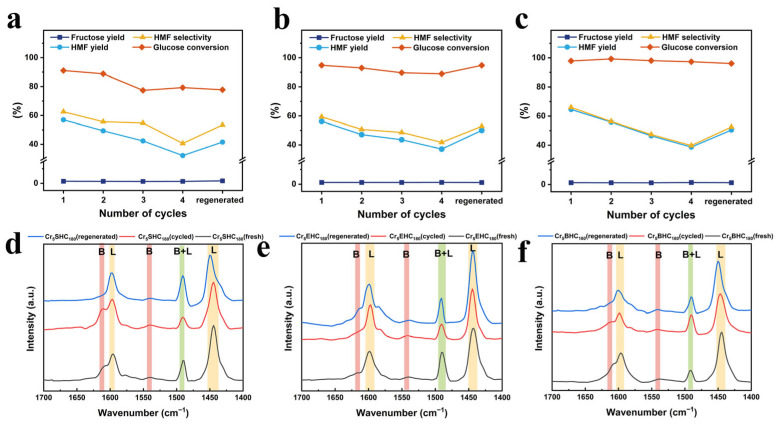
Effect of catalyst recycling on HMF yield and selectivity for (**a**) Cr_5_SHC_180_, (**b**) Cr_5_EHC_180_, and (**c**) Cr_5_BHC_180_. Reaction conditions: 0.5 g glucose, 0.1 g catalyst, and 10 mL DMSO/NaCl_aq_ (9:1, *v*/*v*); (**a**) 170 °C for 3 h, (**b**) 180 °C for 3 h, and (**c**) 170 °C for 5 h. Py-FTIR spectra of the catalysts in fresh, reused, and regenerated states: (**d**) Cr_5_SHC_180_, (**e**) Cr_5_EHC_180_, and (**f**) Cr_5_BHC_180._

- The reusability of the Cr_5_SHC_180_, Cr_5_EHC_180_, and Cr_5_BHC_180_ catalysts was evaluated over four consecutive reaction cycles under the optimal conditions, and the results are presented in Figure 8 and Table 4. After every reaction cycle, the catalysts were recovered through centrifugation, washing, and drying and then were reused in the next cycle. From the results, it is evident that all of the HMF yields showed a trend of slightly decreasing over the cycles, with values of 32.2%, 37.1%, and 38.7% for Cr_5_SHC_180_, Cr_5_EHC_180_ and Cr_5_BHC_180_, respectively, in the last cycle. To investigate the cause of this decline, the spent catalysts were regenerated by aerobic calcination at 300 °C for 1 h. After the regeneration, the HMF yield was recovered to 41.6%, 50.1%, and 50.5%, respectively, suggesting that deactivation is primarily due to the deposition of carbonaceous by-products, such as humins, on active sites.
- Further analyses of deactivation mechanisms included assessments of Cr leaching, the BET surface area, and the pore diameter. After 7 days in water, the content of leached Cr was measured at 1.12, 0.56, and 0.89 mg/L for Cr_5_SHC_180_, Cr_5_EHC_180_, and Cr_5_BHC_180_, respectively (Figure S4). The specific surface areas of the fresh catalysts were 7.84, 20.59, and 19.43 m^2^/g, which were decreased to 5.20, 12.16, and 12.71 m^2^ g^−1^ after four reaction cycles, but then increased significantly to 96.94, 105.43, and 42.97 m^2^ g^−1^ (Table 4, Figure S5) after the generation, indicating a strengthened pore structure.
- The acidic properties were evaluated using Py-FTIR, with the results illustrated in Figure 8d,e, and provided in Table 4. As indicated in the figure, the Lewis acid contents are 28.65, 29.62, and 31.86 μmol g^−1^, and Brønsted acid contents are 2.46, 2.84, and 4.11 μmol g^−1^ for Cr_5_SHC_180_, Cr_5_EHC_180_, and Cr_5_BHC_180_, respectively; after four reaction cycles, the contents were decreased to 19.94, 26.52, and 27.71 μmol g^−1^ for Lewis acid, and to 2.23, 2.92, and 2.87 μmol g^−1^ for Brønsted acid, respectively. Moreover, the total acid content also declined from 31.11–35.97 μmol g^−1^ to 22.19–30.58 μmol g^−1^.
- Following the calcination, the acid content of Cr_5_SHC_180_, Cr_5_EHC_180_, and Cr_5_BHC_180_ was restored to 26.76, 33.87, and 32.15 μmol g^−1^ for the Lewis acid, and to 2.30, 2.00, and 3.46 μmol g^−1^ for the Brønsted acid, giving total acid amounts of 29.06, 35.87, and 35.61 μmol g^−1^, respectively. In conclusion, catalyst deactivation is primarily attributed to the degradation of the pore structure, loss of acidic sites, and humin deposition, all of which are partially reversible and can be restored through regeneration by calcination.

## 4. Discussion

Cr-doped carbonaceous catalysts were successfully prepared by hydrothermal analysis using biomass as the carbon source for the production of HMF from glucose. The incorporation of CrCl_3_ significantly influenced the hydrothermal process, modifying the surface morphology, functional group composition, acid density, acid strength, and the distribution of both the Brønsted acid and Lewis acid sites of the catalysts. These structural and acidic properties are closely linked to the composition of the biomass feedstock, highlighting the critical role of synergistic interactions between catalytically active sites and feedstock components. Among the catalysts studied, the Cr-doped bagasse-based hydrochar (Cr_5_BHC_180_) demonstrated the highest performance, achieving an HMF yield of 64.5% under the optimal conditions (170 °C, 5 h). After four reaction cycles, the catalyst retained an HMF yield of 38.7%, and regeneration by calcination at 300 °C for 1 h restored the yield to 50.5%, confirming its good reusability. The decline in catalytic activity during reuse is primarily attributed to the degradation of the pore structure, loss of acid sites, and humin deposition. This study demonstrates the potential of Cr-doped biomass-derived hydrochar microspheres as efficient and recyclable catalysts for glucose isomerization and HMF production. Furthermore, it provides insights of high value for the design and optimization of metal-doped carbonaceous catalysts for their applications in sustainable biomass conversion.

## Figures and Tables

**Table 1:** XPS fitting results of C 1s, O 1s, and Cr 2p for different biomass-based hydrochar catalysts.

Samples	C 1s			O 1s			Cr 2p
	C=C/ CHx/C-C (%)	C-OH/ C-O-C (%)	-C=O (%)	C-OH/ C-O-C (%)	-C=O (%)	Cr-O (%)	Cr^3+^ (%)
Cr_0_SHC_180_	71.09	23.04	5.86	72.65	27.35	--	--
Cr_5_SHC_180_	74.34	17.76	7.90	50.41	41.08	8.51	100
Cr_0_EHC_180_	50.51	36.64	9.86	89.03	10.97	--	--
Cr_5_EHC_180_	71.35	20.70	7.95	57.21	36.11	6.68	100
Cr_0_BHC_180_	65.15	24.07	10.78	86.92	13.08	--	--
Cr_5_BHC_180_	74.12	17.97	7.91	57.14	35.63	7.24	100

**Table 2:** Specific surface area, average pore size, and carbon yield of different biomass-based hydrochar catalysts.

Samples		BET * Surface Area(m^2^ g^−1^)	Average Pore Size (Diameter, nm)	Carbon Yield (%)
Starch-based	Cr_0_SHC_180_	2.47	19.42	4.61
	Cr_5_SHC_180_	7.84	17.68	18.06
Eucalyptus-based	Cr_0_EHC_180_	2.21	22.73	60.72
	Cr_5_EHC_180_	20.59	25.64	36.36
Bagasse-based	Cr_0_BHC_180_	2.51	20.00	29.12
	Cr_5_BHC_180_	19.43	16.95	23.96

* BET represents Brunauer, Emmett, and Teller.

**Table 3:** Initial potential, acid density, and catalytic performance of hydrochar catalysts.

Samples		Acid Density /mmol g^−1^	Initial Potential /mV	HMF Yield /%	HMF Selectivity /%	Glucose Conversion /%	Fructose Yield /%
Starch-based	Cr_0_SHC_180_	0.14	221	28.3	32.4	87.6	1.4
	Cr_5_SHC_180_	0.20	247	51.7	51.7	100.0	1.2
Eucalyptus-based	Cr_0_EHC_180_	0.1	159	23.1	26.4	87.3	1.5
	Cr_5_EHC_180_	0.18	164	48.8	48.8	100.0	1.3
Bagasse-based	Cr_0_BHC_180_	0.16	225	36.7	40.2	91.3	1.4
	Cr_5_BHC_180_	0.24	265	52.5	52.5	100.0	1.3

**Table 4:** Specific surface area, average pore size, and quantity of acid of hydrochar catalysts.

Samples		BET * Surface Area (m^2^ g^−1^)	Average Pore Size (Diameter, nm)	Brønsted Acid (μmol g^−1^)	Lewis Acid (μmol g^−1^)	Total Acids (μmol g^−1^)
Starch-based	Cr_5_SHC_180_(fresh)	7.84	17.68	2.46	28.65	31.11
	Cr_5_SHC_180_(recycled)	5.20	19.98	2.25	19.94	22.19
	Cr_5_SHC_180_(regenerated)	96.94	9.83	2.30	26.76	29.06
Eucalyptus-based	Cr_5_EHC_180_(fresh)	20.59	25.64	2.84	29.62	32.46
	Cr_5_EHC_180_(recycled)	12.16	28.39	2.92	26.52	29.44
	Cr_5_EHC_180_(regenerated)	105.43	16.98	2.00	33.87	35.87
Bagasse-based	Cr_5_BHC_180_(fresh)	19.43	16.95	4.11	31.86	35.97
	Cr_5_BHC_180_(recycled)	12.71	18.74	2.87	27.71	30.58
	Cr_5_BHC_180_(regenerated)	42.97	15.19	3.46	32.15	35.61

* BET represents Brunauer, Emmett, and Teller.

## Data Availability

The data supporting the reported results can be obtained from the authors upon request.

## Supplementary Materials

The following supporting information can be downloaded at https://www.mdpi.com/article/10.3390/polym17101413/s1: Figure S1. Isothermal N_2_ adsorption and desorption curves of catalysts. (a) Starch-based; (b) eucalyptus-based; (c) bagasse-based. Figure S2. HPLC chromatograms of catalysts. (a) Cr_5_SHC_180_, (b) Cr_5_EHC_180_, (c) Cr_5_BHC_180_; Reaction conditions: 0.5 g glucose; 0.1 g catalyst; DMSO/NaCl_aq_ (9:1, *v*/*v*), (a) 170 °C, 3 h; (b) 180 °C, 3 h; (c) 170 °C, 5 h. Figure S3. HPLC standard curve for HMF. Figure S4: Chromium leaching from catalysts after exposure to water (pH = 7) at 25 °C for seven days. Figure S5: Isothermal N_2_ adsorption and desorption curves of fresh, recycled, and regenerated catalysts: (a) starch-based, (b) eucalyptus-based, (c) bagasse-based.
