# Supplementary material for: Chromium-Doped Biomass-Based Hydrochar-Catalyzed Synthesis of 5-Hydroxymethylfurfural from Glucose
Source: Polymers (Basel). 2025 May 20;17(10):1413. doi: 10.3390/polym17101413 (PMC12114955; doi:10.3390/polym17101413)
Supplement: Supplementary file 1 [file polymers-17-01413-s001.zip › polymers-3643518-supplementary.pdf]

## Supplementary material for

# Chromium-doped biomass-based hydrochar-catalyzed synthe-sis of 5-hydroxymethylfurfural from glucose

**Huimin Gao, Wei Mao, Pize Xiao, Chutong Ling, Zhiming Wu, Jinghong Zhou ***

Guangxi Key Laboratory of Clean Pulp & Papermaking and Pollution Control, School of Light Industrial and Food Engineering, Guangxi University, Nanning 530004, China

* Correspondence: jhzhoudou@gxu.edu.cn

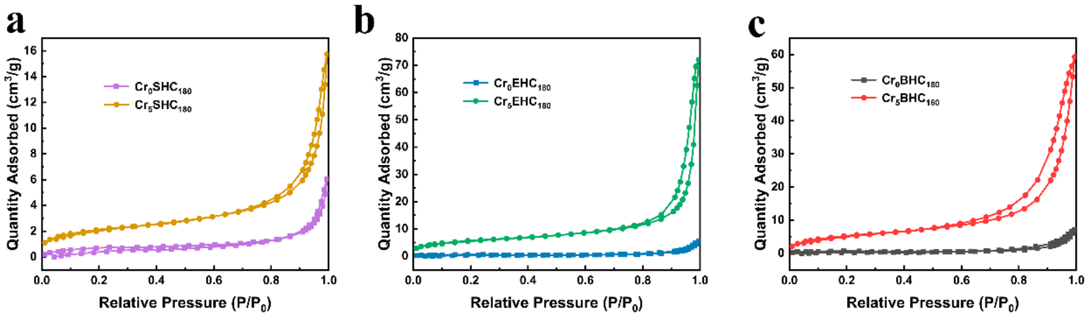


**Figure S1.** Isothermal $N_2$ adsorption and desorption curves of catalysts. (a) Starch-based; (b) Eucalyptus-based; (c) Bagasse-based.

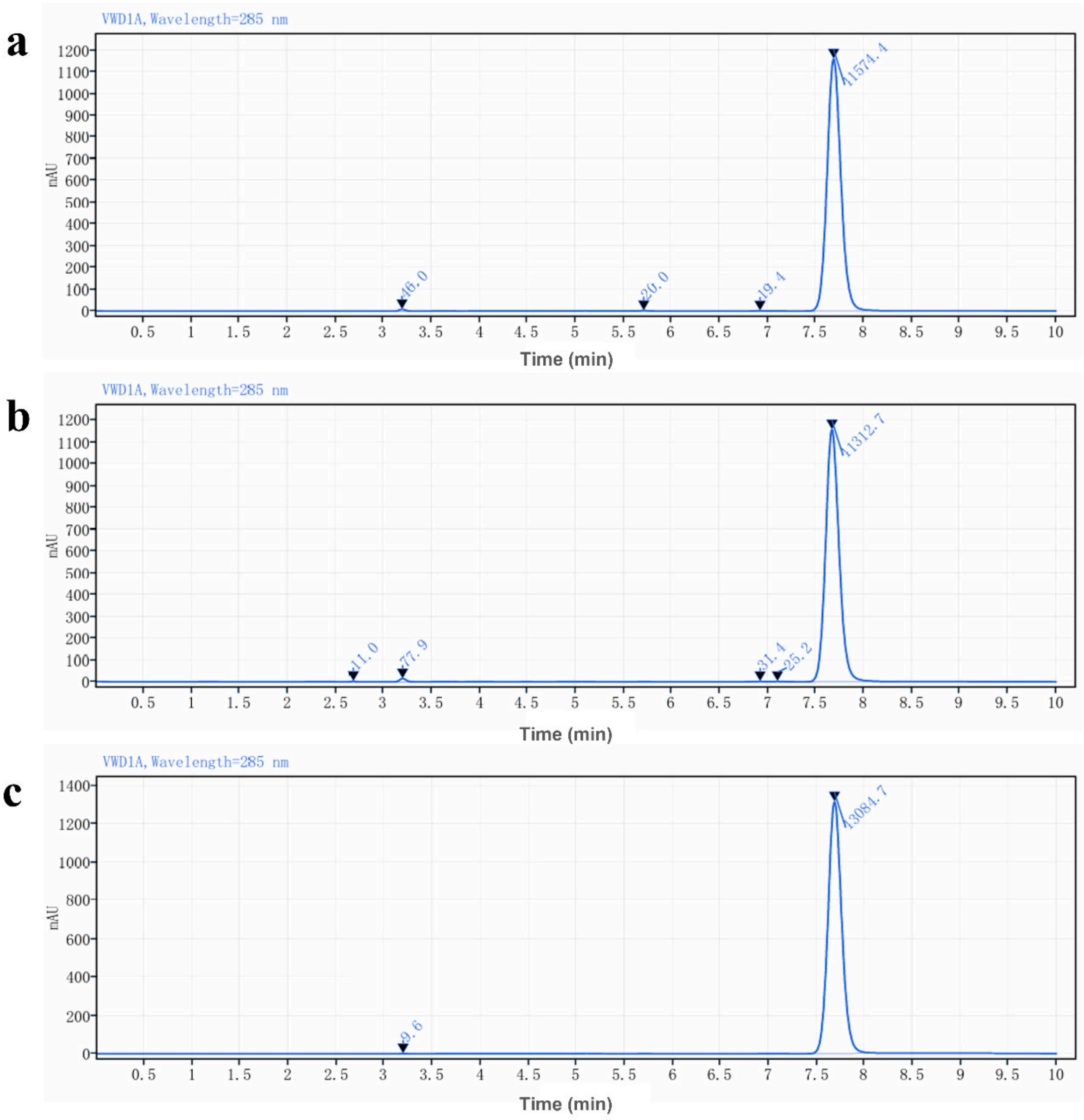


**Figure S2.** HPLC chromatograms of the reaction mixtures obtained using (a) $Cr_5SHC_{180}$, (b) $Cr_5EHC_{180}$, and (c) $Cr_5BHC_{180}$ catalysts. Reaction conditions: 0.5 g glucose, 0.1 g catalyst, 10 mL DMSO/$NaCl_{aq}$ (9:1, v/v); (a) 170 °C for 3h, (b) 180 °C for 3h, and (c) 170 °C for 5h

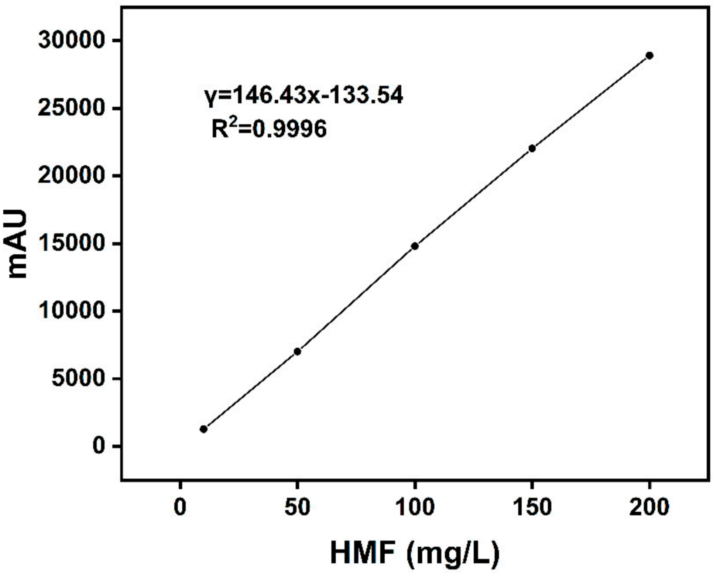


**Figure S3.** HPLC standard curve for HMF.

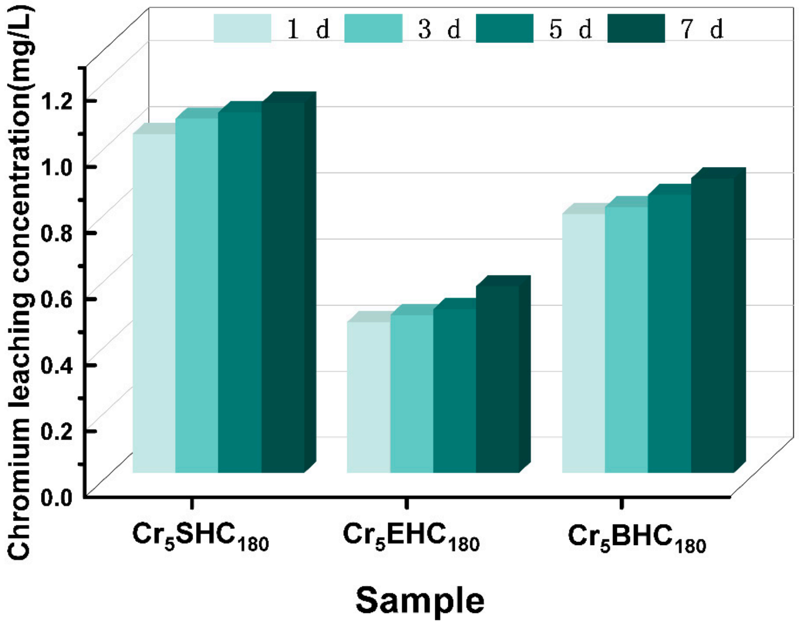


**Figure S4.** Chromium leaching from catalysts after exposure to water (pH=7) at 25 °C for seven days.

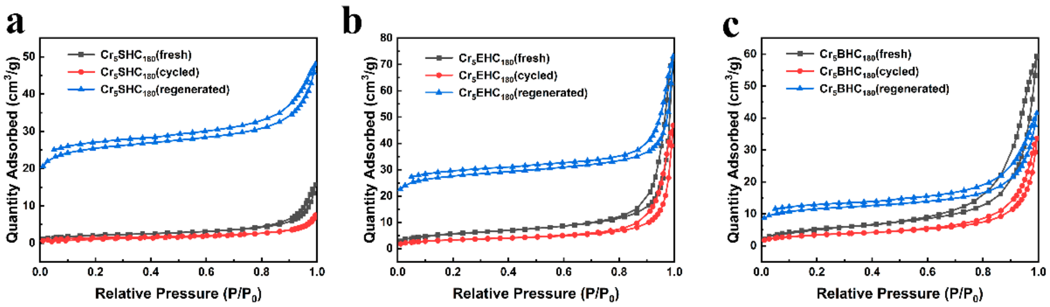


**Figure S5.** Isothermal $N_2$ adsorption and desorption curves of fresh, cycled and regenerated catalysts. (a) Starch-based; (b) Eucalyptus-based; (c) Bagasse-based.
